# The therapeutic effects of natural organosulfur compounds on atherosclerosis and their potential mechanisms: a comprehensive review

**DOI:** 10.3389/fcvm.2025.1599154

**Published:** 2025-07-01

**Authors:** Yuanyuan Tang, De Lv, Yijing Tao, Juan Wang

**Affiliations:** 1 Department of Pharmacy, Hospital of Chengdu University of Traditional Chinese Medicine, Chengdu, China; 2 School of Pharmacy, Institute of Material Medica, North Sichuan Medical College, Nanchong, China; 3 Department of Endocrinology, Hospital of Chengdu University of Traditional Chinese Medicine, Chengdu, China; 4 Department of Cardiology, Changshu Hospital Affiliated to Soochow University, Changshu No.1 People’s Hospital, Changshu, China

**Keywords:** organosulfur compounds, garlic, atherosclerosis, lipid metabolism, oxidative stress, anti-Inflammatory agents, endothelial protection, PCSK9 inhibitors

## Abstract

Atherosclerosis (AS) is a major cause of cardiovascular disease morbidity and mortality, characterized by lipid accumulation, oxidative stress, and chronic inflammation. Natural organic sulfur compounds (OSCs), especially those derived from garlic (Allium sativum), have therapeutic value in slowing the course of AS. We systematically evaluate the mechanisms by which OSCs exert their anti-atherogenic effects, focusing on lipid metabolism regulation, antioxidant defense, anti-inflammatory responses, endothelial protection, and antibacterial activity. Key signaling pathways, including Nrf2/ARE, RhoA/ROCK, AMPK/SREBP-1c/SREBP-2, and PCSK9-LDLR, are highlighted as critical mediators of these effects. Preclinical and clinical investigations show that OSCs significantly reduce plasma cholesterol, suppress oxidative stress, and attenuate inflammatory cascades. However, challenges such as variable bioavailability and the absence of standardized formulations limit their clinical application. Future research should focus on clinical trials to establish efficacy, improve bioavailability, and create standardized formulations of OSCs for cardiovascular disease prevention and management.

## 1 Introduction

Cardiovascular disease (CVD) persists as the predominant cause of mortality across all disease categories in China. Notably, since 2020, CVD mortality rates in rural regions have consistently exceeded those in urban areas. Statistical data from 2024 indicate that the age-standardized CVD mortality rate reached 298.42 per 100,000 population in rural areas, compared to 264.84 per 100,000 in urban areas (1). This epidemiological pattern underscores the significant public health burden imposed by CVD, with atherosclerosis (AS) constituting the fundamental pathological mechanism underlying most CVD cases (2).

The development and progression of AS are intrinsically associated with plasma lipid levels, particularly total cholesterol (TC) and triglycerides (TG) concentrations.

**Figure 1:**
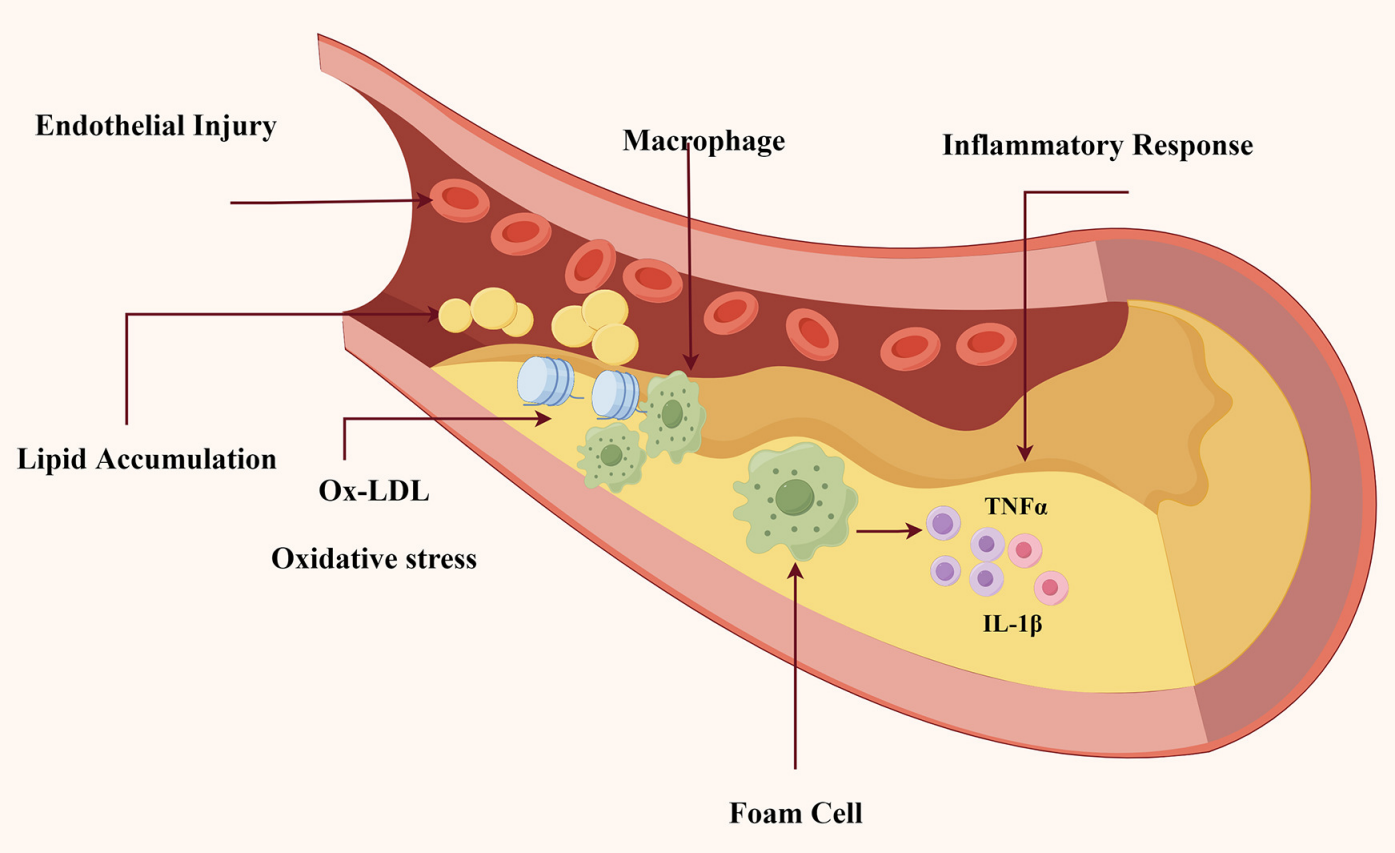
The underlying pathogenesis in atherosclerosis. As indicated in Figure. This schematic diagram depicts the essential pathophysiological processes involved in the beginning and development of AS: (1) Injury to endothelial cells initiates atherosclerotic plaque formation; (2) Lipid accumulation; and (3) Ox-LDL infiltration and macrophage recruitment; (4) Macrophages transform into foam cells and release pro-inflammatory cytokines (TNF-α, IL-1β), leading to persistent inflammation and plaque instability.

Pathophysiological studies have demonstrated that elevated plasma lipid levels facilitate the infiltration and deposition of lipids within the arterial intima, subsequently triggering smooth muscle cell proliferation and foam cell formation—the hallmark cellular events in atherogenesis (3). Contemporary research has identified multiple risk factors contributing to AS pathogenesis, including but not limited to hypertension, diabetes mellitus, hyperlipidemia, obesity, tobacco use, and microbial infections. Recent investigations, illustrated in Figure 1, show that the complicated pathophysiology of AS involves numerous connected pathways, including oxidative stress, lipid accumulation, inflammatory response, and endothelial injury (4–6).

Natural organosulfur compounds (OSCs), a class of bioactive molecules with both hydrophilic and lipophilic properties, are predominantly distributed in plant species belonging to the Cruciferae and Liliaceae families (7). Among these, garlic (Allium sativum) has attracted particular attention from the scientific community due to its rich OSC content. Biochemical analyses have identified over 30 distinct OSCs in garlic, with the majority being enzymatically derived from L-cysteine sulfoxides and γ-glutamyl-L-cysteine peptides through various biosynthetic pathways (8).

Freeze-dried preparations of wild garlic (Allium ursinum) cloves have been shown to contain a diverse array of OSCs, with alliin being the predominant form stored in the cytoplasmic compartment of intact cells. The mechanical disruption of garlic cloves triggers the enzymatic conversion of alliin by alliinase, leading to the formation of allicin, which is responsible for the characteristic pungent aroma (9). Historical records from traditional Chinese medicine document the use of raw garlic for the prevention and treatment of atherosclerotic conditions.

Modern epidemiological evidence supports this traditional knowledge, demonstrating an inverse correlation between the consumption of fruits and vegetables and cardiovascular disease risk (10, 11). This relationship has been further substantiated by a longitudinal study involving 1,226 Australian women aged over 70 years, which revealed that increased consumption of cruciferous and liliaceous vegetables (≥75 g/day) was associated with reduced mortality from atherosclerotic cardiovascular disease over a 15-year follow-up period (12).

This review synthesizes current scientific literature to investigate the anti-atherosclerotic properties of OSCs and their underlying molecular mechanisms. Meanwhile, the purpose of this review is to summarize the current evidence on OSC-mediated anti-atherosclerotic mechanisms, addressing gaps in clinical translation and proposing future research directions.

## 2 Sources and classification of natural OSCs

**Figure 2:**
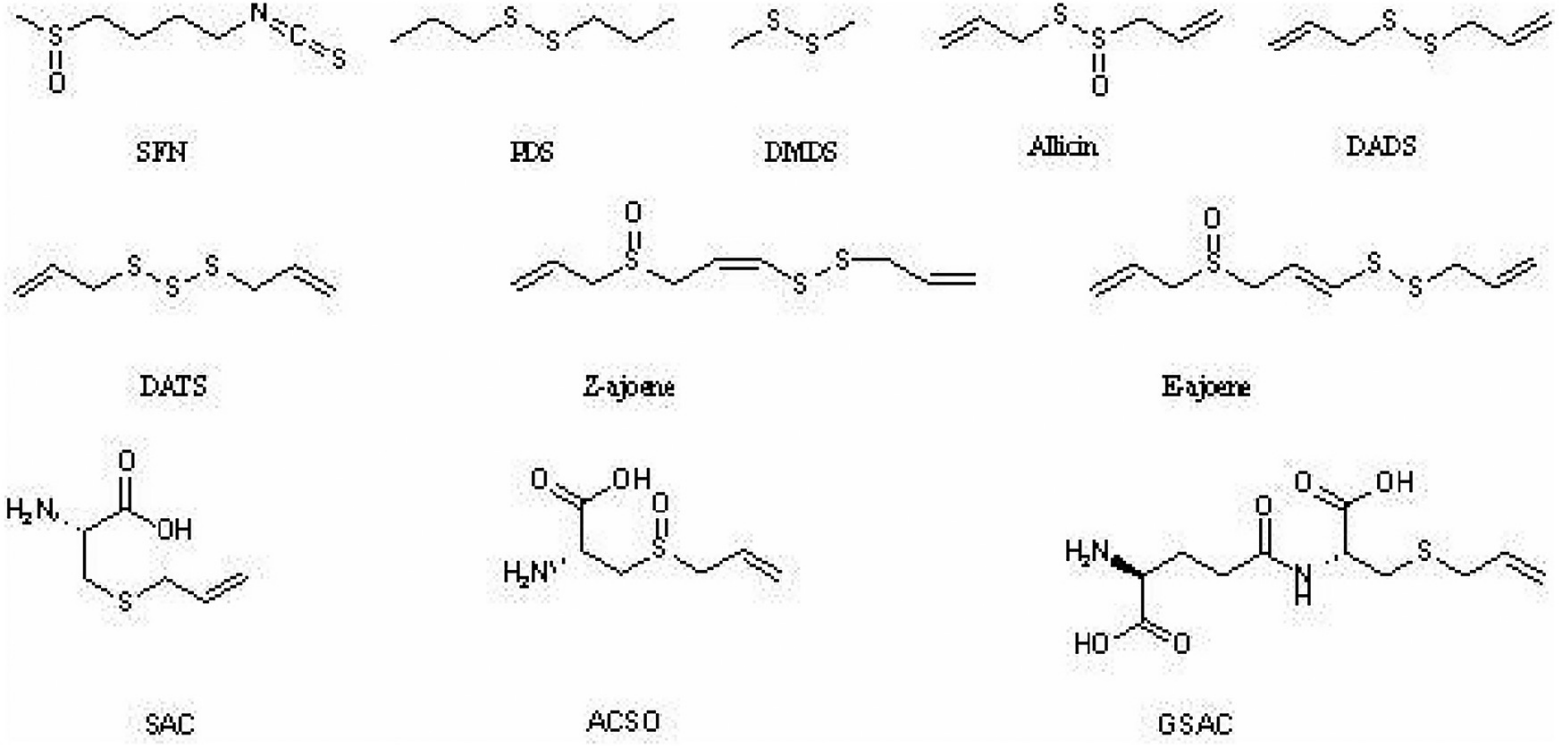
Chemical structure of natural organosulfur compounds. Figure shows the chemical structure of lipophilic (fat-soluble) and hydrophilic (water-soluble) natural organosulfur compounds with anti-atherosclerotic properties: (1) Lipophilic OSCs: sulforaphane (SFN), allicin, dimethyl disulfide (DMDS), propyl disulfide (PDS), diallyl disulfide (DADS), diallyl trisulfide (DATS), cis-ajoene (Z-ajoene), and trans-ajoene (E-ajoene); (2) Hydrophilic OSCs: S-allyl-L-cysteine (SAC), S-allyl-L-cysteine-sulfoxide (ACSO), and *γ*-L-glutamyl-S-allyl-L-cysteine (GSAC).

Organosulfur compounds (OSCs) represent a distinct class of phytochemicals characterized by the presence of sulfur-containing functional groups within their molecular architecture. These bioactive compounds are predominantly concentrated in garlic bulbs, with particularly high levels being detected in this plant species (13). Although garlic is the primary source of OSCs, other important dietary sources include the Cruciferae (broccoli, Brussels sprouts) and Liliaceae (onions, leeks) families. For instance: Broccoli contains sulforaphane (SFN), a strong lipophilic OSC with anti-inflammatory as well as antioxidant properties. Similarly, onions contain allicin, diallyl disulfide (DADS), diallyl trisulfide (DATS), and other sulfur compounds that have been associated with cardiovascular benefits to health. Based on their physicochemical properties, OSCs can be categorized into two primary groups: lipophilic (fat-soluble) and hydrophilic (water-soluble) compounds, as shown in Figure 2.

The lipophilic fraction of OSCs with demonstrated anti-atherosclerotic activity includes several well-characterized compounds: sulforaphane (SFN), allicin, dimethyl disulfide (DMDS), propyl disulfide (PDS), diallyl disulfide (DADS), diallyl trisulfide (DATS), cis-ajoene (Z-ajoene), and trans-ajoene (E-ajoene). Conversely, the hydrophilic OSC group with anti-atherogenic properties comprises S-allyl-L-cysteine (SAC), S-allyl-L-cysteine-sulfoxide (ACSO), and γ-L-glutamyl-S-allyl-L-cysteine (GSAC). Extensive research has been conducted on the anti-atherogenic mechanisms of lipophilic OSCs, particularly allicin and SFN. Similarly, water-soluble OSCs, including SAC, S-ethyl-L-cysteine (SEC), ACSO, and GSAC, have demonstrated significant pharmacological potential through multiple mechanisms, such as lipid-lowering, antioxidant, and anti-inflammatory effects (14, 15).

## 3 Biological functions of natural organosulfur compounds in anti-atherosclerosis

### 3.1 Lipid-lowering activity

Dyslipidemia, characterized by abnormal elevations in plasma lipid concentrations, particularly total cholesterol (TC) and low-density lipoprotein (LDL), represents a well-established risk factor for the development of atherosclerosis (AS) (16, 17). This pathological condition is specifically defined by increased LDL concentrations coupled with decreased high-density lipoprotein (HDL) levels. Recent pharmacological studies have demonstrated that garlic oil nanoemulsion exhibits significant lipid-lowering effects in hyperlipidemic Wistar rat models (18, 19).

The cholesterol-reducing properties of aged garlic extract (AGE) have been consistently observed in both human clinical trials and animal studies. Mechanistic investigations using Sprague-Dawley (SD) rats have revealed that this hypocholesterolemic effect is primarily mediated through the inhibition of hepatic cholesterol biosynthesis pathways (20). *in vitro* experimental data have further demonstrated that water-soluble OSCs, particularly SAC, effectively suppress cholesterol synthesis (21). The integration of *in vitro* and *in vivo* findings suggests that SAC may represent the primary bioactive component responsible for AGE-mediated plasma cholesterol reduction (22, 23).

Furthermore, allicin has been shown to ameliorate lipid metabolic disorders in HepG2 cells induced by 1,3-dichloropropanol exposure, as evidenced by significant reductions in both TG and TC levels (24). The molecular mechanisms underlying these effects appear to involve the modulation of key signaling pathways, including AMPK-SREBPs and PKA-CREB (25). Complementary research by Ma et al. (26) has revealed that DADS suppresses lipoprotein (a) [Lp(a)] expression in HepG2 cells through the MEK1-ERK1/2-ELK-1 signaling cascade.

Current research indicates that OSCs like allicin and DADS modulate the lipid metabolic process of HepG2 cells via pathways such as AMPK-SREBPs and MEK1-ERK1/2-ELK-1, resulting in effective lipid-lowering effects. Clinical investigations have demonstrated the cholesterol-lowering effects of AGE, and *in vivo* and *in vitro* experiments have confirmed that SAC is the fundamental bioactive component responsible for AGE-mediated plasma cholesterol reduction. The absence of standardized formulations and variable bioavailability of OSCs, however, makes OSCs difficult to use in treating AS. To improve practical applicability, future research should focus on nanocarrier-based drug delivery technologies and statin combination therapy.

### 3.2 Antioxidant activity

Oxidative stress represents a state of persistent cellular oxidative damage in biological systems, resulting from either excessive production of reactive oxygen species (ROS) or diminished antioxidant defense capacity. Clinical studies have demonstrated that individuals with chronic metabolic disorders, including AS, hypertension, hyperlipidemia, and diabetes mellitus, exhibit increased susceptibility to AS progression when experiencing systemic oxidative stress (27, 28). These findings underscore the pivotal role of oxidative stress in the pathogenesis of AS.

Under normal physiological conditions, ROS serve essential signaling functions at regulated concentrations. However, oxidative stress ensues when ROS production exceeds the cellular antioxidant capacity. Experimental evidence indicates that allicin (0.3–10 μmol/L) confers concentration-dependent protection against H2O2-induced cytotoxicity in H9C2 cardiomyocytes (29). Although allicin demonstrates limited 2,2-diphenyl-1-picrylhydrazyl (DPPH) radical scavenging activity, its cytoprotective effects appear to be mediated through the reduction of intracellular ROS accumulation, as proposed by Ma et al. (30). Complementary research by Wang et al. has reported that water-soluble OSCs, specifically SAC, ACSO, and GSAC, exhibit significant antioxidant properties through DPPH radical scavenging and ferrous ion chelation mechanisms (31).

Pathophysiological investigations have revealed that under hyperoxic conditions, LDL undergoes oxidation to form oxidized LDL (ox-LDL), which exerts cytotoxic effects on vascular endothelial cells. The resulting endothelial dysfunction subsequently accelerates LDL oxidation, thereby establishing a vicious cycle that promotes atherogenesis (32, 33). Recent research have identified that DADS and DATS protect endothelial nitric oxide synthase (eNOS) activity against ox-LDL-induced damage, potentially through modulation of the PI3 K/PKB signaling pathway and inhibition of eNOS degradation (34).

Chronic hyperglycemia-induced oxidative stress represents a significant contributor to endothelial dysfunction and atherogenesis (35). Preclinical studies have shown that DATS administration significantly reduces malondialdehyde (MDA) and ROS levels while enhancing mitochondrial superoxide dismutase (SOD) and glutathione peroxidase (GSH-Px) activity (36). Furthermore, DATS has been shown to improve mitochondrial respiratory function, suggesting its potential therapeutic application in oxidative stress-related pathologies, including AS, diabetes mellitus, and neurodegenerative disorders.

In summary, OSCs reduce oxidative stress through a variety of approaches, including ROS suppression, DPPH radical scavenging, ferrous ion chelation, and upregulation of endogenous antioxidant enzymes such as SOD and GSH-Px. In particular, DADS and DATS protect endothelial cells from ox-LDL-induced damage. A suitable dose of antioxidant will be required for future OSCs investigation.

### 3.3 Anti-inflammatory activity

The understanding of AS pathogenesis has evolved significantly from the traditional view of passive lipid accumulation in arterial walls to the current recognition of AS as a chronic inflammatory disease. This paradigm shift is supported by the identification of various inflammatory mediators and immune cells that contribute to the development and progression of atherosclerotic plaques (37).

Emerging evidence suggests that SAC exhibits anti-inflammatory properties through multiple mechanisms. Experimental studies have demonstrated that SAC may prevent skeletal muscle atrophy by modulating the expression of specific pro-inflammatory factors (38). Furthermore, SAC has been shown to mitigate lipopolysaccharide (LPS)-induced inflammation in 3T3-L1 adipocytes through upregulation of anti-inflammatory gene expression and metabolic modulation (39, 40).

The anti-inflammatory effects of OSCs extend beyond SAC. Research has revealed that (Z, E)-ajoene and its sulfonylated derivatives inhibit LPS-induced production of nitric oxide (NO) and prostaglandin E2 (PGE2) by suppressing inducible nitric oxide synthase (iNOS) and cyclooxygenase-2 (COX-2) expression (41). This effect is mediated through the inhibition of NF-*κ*B activation and reduction of p38 and ERK phosphorylation. Similarly, DATS has been shown to attenuate cytokine production and inflammatory mediator release, including interleukin-6 (IL-6), interleukin-10 (IL-10), tumor necrosis factor-α (TNF-α), iNOS, and COX-2, through the blockade of NF-*κ*B and MAPK signaling pathways (42).

Complementary studies by Chu et al. using an LPS-induced RAW 264.7 macrophage model demonstrated that DADS, DMDS, and propyl disulfide (PDS) effectively suppress NO and PGE2 synthesis, thereby limiting LPS-induced inflammatory responses (43, 44). Collectively, both *in vitro* and *in vivo* studies indicate that OSCs exert their anti-inflammatory effects primarily through the inhibition of NF-*κ*B and MAPK signaling pathways, as well as the suppression of inflammatory mediators such as IL-6, IL-10, and TNF-α.

### 3.4 Protective effect on endothelial cells

Vascular endothelial cells play a crucial role in maintaining vascular homeostasis by regulating vascular smooth muscle cell proliferation and migration, while simultaneously modulating inflammatory responses and thrombotic processes. These protective functions are essential for maintaining vascular integrity (45). Disruption of endothelial function initiates a cascade of pathophysiological events that contribute to the development and progression of AS.

Experimental studies have demonstrated that allicin exerts protective effects against oxidized low-density lipoprotein (ox-LDL)-induced injury in human umbilical vein endothelial cells (HUVECs) through the inhibition of apoptotic pathways and attenuation of oxidative stress. Clinical investigations by Liu et al. have further revealed that allicin administration significantly reduces plasma homocysteine (Hcy) levels in patients with coronary heart disease (46). Elevated Hcy concentrations, particularly in the range of 10–1,000 μmol/L, have been shown to negatively correlate with HUVEC viability, with concentrations reaching 1,000 μmol/L resulting in significant endothelial cell dysfunction and apoptosis (47, 48).

DATS has emerged as a potential therapeutic agent through its ability to release hydrogen sulfide (H_2_S), a gaseous signaling molecule. Under physiological conditions, DATS-mediated H_2_S release enhances vascular responses following ischemic events, primarily through increased bioavailability of H_2_S and subsequent activation of endothelial nitric oxide synthase (eNOS) (49, 50). As the third endogenous gasotransmitter following nitric oxide (NO) and carbon monoxide (CO), H_2_S has been demonstrated to promote endothelial cell proliferation and migration at physiological concentrations (10–20 μmol/L). However, this stimulatory effect transitions to inhibition at elevated concentrations (500–1,000 μmol/L) (51, 52).

In conclusion, OSCs, such as allicin and DATS, protect vascular endothelial cells via three different processes: (1) suppression of apoptotic pathways, (2) lowering of plasma Hcy levels, and (3) H_2_S-mediated activation of eNOS function. Future research should focus on recognizing the therapeutic potential of OSCs-derived H_2_S donors in endothelial protection and their function in preventing AS development.

### 3.5 Antibacterial activity

According to epidemiological studies, a variety of bacterial and viral infections have been causally linked to the development of AS. Lin et al. identified pathogenic microorganisms in both human atherosclerotic tissues and animal models of AS (53).

Porphyromonas gingivalis (P. gingivalis), a key pathogen implicated in periodontitis, has been demonstrated to promote lipid accumulation in the vascular wall and contribute to the development of AS through multiple molecular mechanisms (54, 55). *in vitro* studies investigating the antibacterial efficacy of allicin against oral pathogens associated with dental caries and periodontitis have revealed that allicin, at a concentration of 2,400 μg/ml, significantly inhibits the growth of P. gingivalis (56).

Quorum sensing (QS), a bacterial cell-to-cell communication system, represents a promising target for anti-pathogenic interventions. Recent research has identified that Z-ajoene, a compound containing disulfide linkages, exhibits significant QS inhibitory properties (57). Moreover, DADS, another organosulfur compound, has demonstrated potent antibacterial activity against clinically relevant pathogens, including Staphylococcus aureus, Pseudomonas aeruginosa, and Escherichia coli (58). These findings highlight the potential of OSCs as therapeutic agents targeting both bacterial growth and virulence mechanisms.

In overall, DADS has broad-spectrum antibacterial activity, while Z-ajoene has considerable QS inhibitory properties, and allicin has a significant antibacterial effect on P. gingivalis, a periodontal pathogen implicated in AS pathogenesis through chronic infection. However, allicin's poor *in vivo* stability restricts its clinical application. The combination of stable analogues (for instance, allicin-loaded nanoparticles) and QS inhibitors may enhance pharmacological efficacy.

Up to now, the anti-atherosclerotic effects of OSCs, including their classification (lipophilic/hydrophilic), biological functions, mechanisms, study models, and key findings have been summarized in Table 1.

**Table 1:** Anti-atherosclerotic effects of natural organosulfur compounds.

Compound	Class	Biological function	Mechanism of action	Study model	Key findings	Reference
Allicin	Lipophilic	Lipid-lowering	Inhibits hepatic cholesterol synthesis via AMPK-SREBP pathway	HepG2 cells, SD rats	Reduces TC, TG levels; activates AMPK	(24, 25)
SAC (S-allyl-L-cysteine)	Hydrophilic	Lipid-lowering	Suppresses cholesterol synthesis	*in vitro*, clinical trials	Primary bioactive component in AGE for cholesterol reduction	(21–23)
DADS (diallyl disulfide)	Lipophilic	Lipid-lowering	Suppresses Lp(a) via MEK1-ERK1/2-ELK-1 pathway	HepG2 cells	Reduces lipoprotein(a) expression	(26)
DATS (diallyl trisulfide)	Lipophilic	Antioxidant	Increases SOD, GSH-Px; reduces MDA, ROS	Preclinical models	Protects against ox-LDL-induced endothelial damage	(34, 36)
Allicin	Lipophilic	Antioxidant	Activates Nrf2/ARE pathway	H9C2 cells	Reduces H2O2-induced oxidative stress	(29, 30)
SAC	Hydrophilic	Antioxidant	DPPH radical scavenging, ferrous ion chelation	*in vitro*	Direct antioxidant activity	(31)
Z-ajoene	Lipophilic	Antibacterial	Quorum sensing inhibition	*in vitro*	Inhibits bacterial communication	(57)
Allicin	Lipophilic	Antibacterial	Growth inhibition of P. gingivalis	*in vitro*	2,400 μg/ml inhibits periodontal pathogen	(56)
DADS	Lipophilic	Antibacterial	Broad-spectrum activity	*in vitro*	Effective against S. aureus, P. aeruginosa	(58)
Alliin	Hydrophilic	Lipid regulation	Activates AMPK/SREBP-2/LDLR pathway	HepG2 cells, SD rats	Reduces plasma LDL-C levels	(73, 77)
SFN (sulforaphane)	Lipophilic	Anti-inflammatory	Inhibits RhoA/ROCK/NF-*κ*B pathway	Endothelial cells	Reduces ICAM-1 expression	(67, 68)

## 4 Associated signaling pathways of natural organosulfur compounds in anti-atherosclerosis

### 4.1 The Nrf2/ARE signaling pathway

The Nrf2/ARE signaling pathway is crucial in the prevention and treatment of various diseases, including anti-atherosclerosis, anti-inflammation, and anti-oxidation (59). Nuclear factor erythroid 2-related factor 2 (Nrf2) is a key activator of the antioxidant responsive element (ARE) (60, 61). ARE is located in the upstream regulatory region of several protective genes and functions primarily by inducing the production of protective proteins through the coordination of these genes, thereby mitigating cellular damage caused by ROS.

Allicin could reduce lipopolysaccharide (LPS)-induced vascular injury in HUVECs (62). The protective mechanism of allicin is closely associated with its anti-oxidative stress and anti-inflammatory properties. Specifically, allicin was found to activate Nrf2, thereby protecting against LPS-induced vascular injury and attenuating vascular inflammation (63). Furthermore, studies have shown that organosulfur compounds such as SAC can directly activate Nrf2 to exert antioxidant effects (64). Other organic sulfides, such as alliin, competitively inhibit the expression of ARE genes due to the presence of cysteine groups in their structure, which interact with Nrf2, thereby playing an antioxidant role.

### 4.2 The rhoA/ROCK signaling pathway

The RhoA/ROCK signaling pathway is integral to both the initial stages of AS, particularly endothelial cell dysfunction, and the later stages involving the formation and rupture of atherosclerotic plaques (65). This pathway regulates the expression of endothelial nitric oxide synthase (eNOS), which decreases with increased eNOS mRNA stability, thereby protecting endothelial function from damage. Additionally, the RhoA/ROCK pathway enhances endothelial permeability by directly phosphorylating myosin light chain (MLC) or inhibiting myosin light chain phosphatase (MLCP) activity (66). It also mediates the proliferation and migration of vascular smooth muscle cells (VSMCs).

SFN inhibits the NF-*κ*B DNA-binding protein and downregulates TNF-α-mediated intercellular adhesion molecule-1 (ICAM-1) expression in endothelial cells by inhibiting the RhoA/ROCK/NF-*κ*B signaling pathway (67). Consequently, SFN plays a multifaceted role in inhibiting inflammation within atherosclerotic lesions, highlighting its potential in the prevention and treatment of AS and other inflammatory diseases (68).

### 4.3 The AMPK/SREBP-1c/SREBP-2 signaling pathway

Adenosine 5'-monophosphate (AMP)-activated protein kinase (AMPK) is phosphorylated and activated by upstream kinases under various physiological and pathological conditions (69, 70). AMPK activation inhibits ROS production induced by mitochondrial dysfunction and endoplasmic reticulum stress, reduces the generation of pro-inflammatory factors caused by dyslipidemia and hyperglycemia, and prevents vascular endothelial dysfunction by increasing nitric oxide (NO) bioavailability (71, 72). Therefore, AMPK activation has emerged as a significant target for the prevention and treatment of AS.

Alliin can activate AMPK and regulate the expression of its downstream targets, sterol regulatory element-binding protein-1c (SREBP-1c) and sterol regulatory element-binding protein-2 (SREBP-2), thereby reducing triglyceride and cholesterol accumulation in HepG2 cells induced by 1,3-dichloro-2-propanol (1,3-DCP) (73). SREBP-1c, primarily expressed in the liver, intestines, and adipose tissue, regulates triglyceride and cholesterol synthesis. Overactivation of SREBP-1c can lead to metabolic diseases such as obesity and fatty liver. Sangeetha et al. confirmed that alliin inhibits SREBP-1c expression, reducing lipid accumulation in hepatocytes induced by free fatty acids (74). SREBP-2 is a key transcription factor responsible for regulating cholesterol synthesis. When cholesterol levels in the endoplasmic reticulum decrease, SREBP-2 is activated, increasing low-density lipoprotein receptor (LDLR) synthesis in the Golgi apparatus and promoting cholesterol uptake into cells, thereby reducing plasma LDL-cholesterol levels (75, 76). Studies have shown that alliin significantly reduces plasma LDL-cholesterol levels in high-fat diet-fed Sprague-Dawley (SD) rats by activating the SREBP-2/LDLR pathway (77).

### 4.4 The PCSK9-LDLR signaling pathway

Proprotein convertase subtilisin/kexin type 9 (PCSK9), identified in the early 2000s, is a critical regulator of LDL-cholesterol metabolism due to its ability to degrade LDL receptors (LDLRs) and clear circulating LDL-cholesterol (78, 79). PCSK9 degrades LDLR through both extracellular and intracellular pathways. In the extracellular pathway, PCSK9 binds to LDLR on the cell surface, forming a PCSK9-LDLR complex that is directed to lysosomes for degradation (80). In the intracellular pathway, PCSK9 binds directly to LDLR in the Golgi apparatus, inducing its degradation. Dyslipidemia, particularly elevated LDL-cholesterol levels, is a primary factor in the development and progression of AS. The level of LDL-cholesterol in the body is mainly determined by the number of LDLRs in the liver and the activity of PCSK9. Increased PCSK9 activity promotes LDLR degradation, leading to higher circulating LDL-cholesterol levels. A European study found that only 43% of patients using statins achieved the goal of reducing LDL-cholesterol levels (81). Consequently, PCSK9 inhibitors have been extensively studied as a novel therapeutic target for lowering LDL-cholesterol levels in patients with hypercholesterolemia (82).

**Figure 3:**
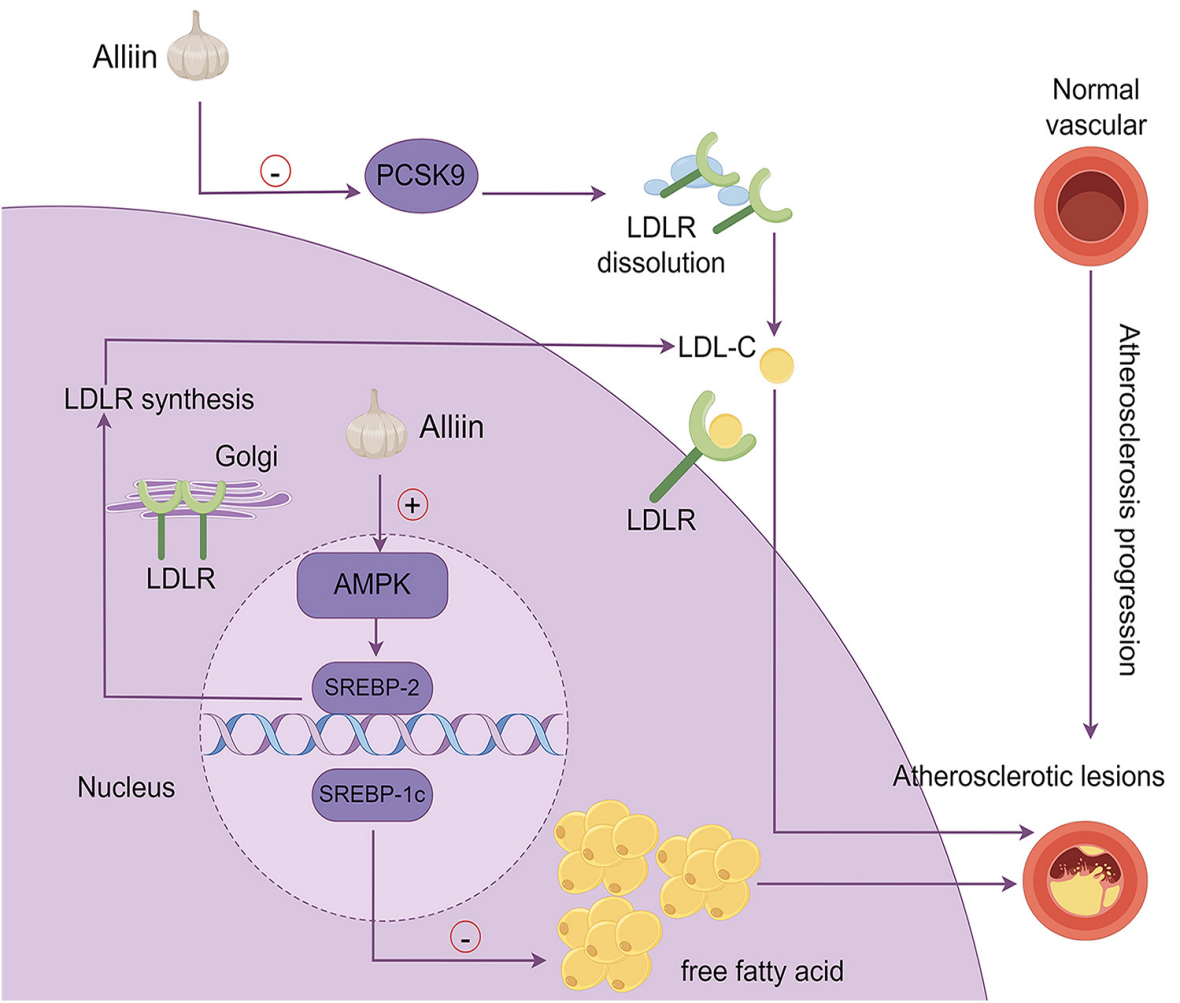
The mechanism by which alliin exerts its anti-aS effects. This diagram summarizes the molecular pathways through which alliin mitigates atherosclerosis: (1) PCSK9-LDLR pathway: alliin suppresses PCSK9 activity, preventing LDL receptor dissolution and lowering circulating LDL-cholesterol; (2) AMPK/SREBP-2 pathway: alliin activates AMPK, which regulates SREBP-2, leading to increased LDLR synthesis in the Golgi apparatus and enhanced cellular cholesterol uptake, thereby reducing plasma LDL-cholesterol levels; (3) AMPK/SREBP-1c pathway: alliin suppresses SREBP-1c expression, reducing lipid accumulation in hepatocytes induced by free fatty acids.

Ahmad et al. investigated the components of proprotein convertase subtilisin/kexin 9 - garlic-derived organic sulfides (PCSK9-OSCs) and discovered that alliin is the most potent inhibitor of PCSK9 activity (83). Since PCSK9 is known to degrades LDLR, the discovery of alliin's inhibitory effect on PCSK9 activity implies that it has the potential to lower circulating LDL cholesterol levels. This is especially important for patients who are intolerant of statins or do not achieve adequate cholesterol reduction with ezetimibe combination therapy. The anti-AS effects of alliin, mediated through the AMPK/SREBP-1c/SREBP-2 signaling pathway and the PCSK9-LDLR signaling pathways, are illustrated in Figure 3.

**Figure 4:**
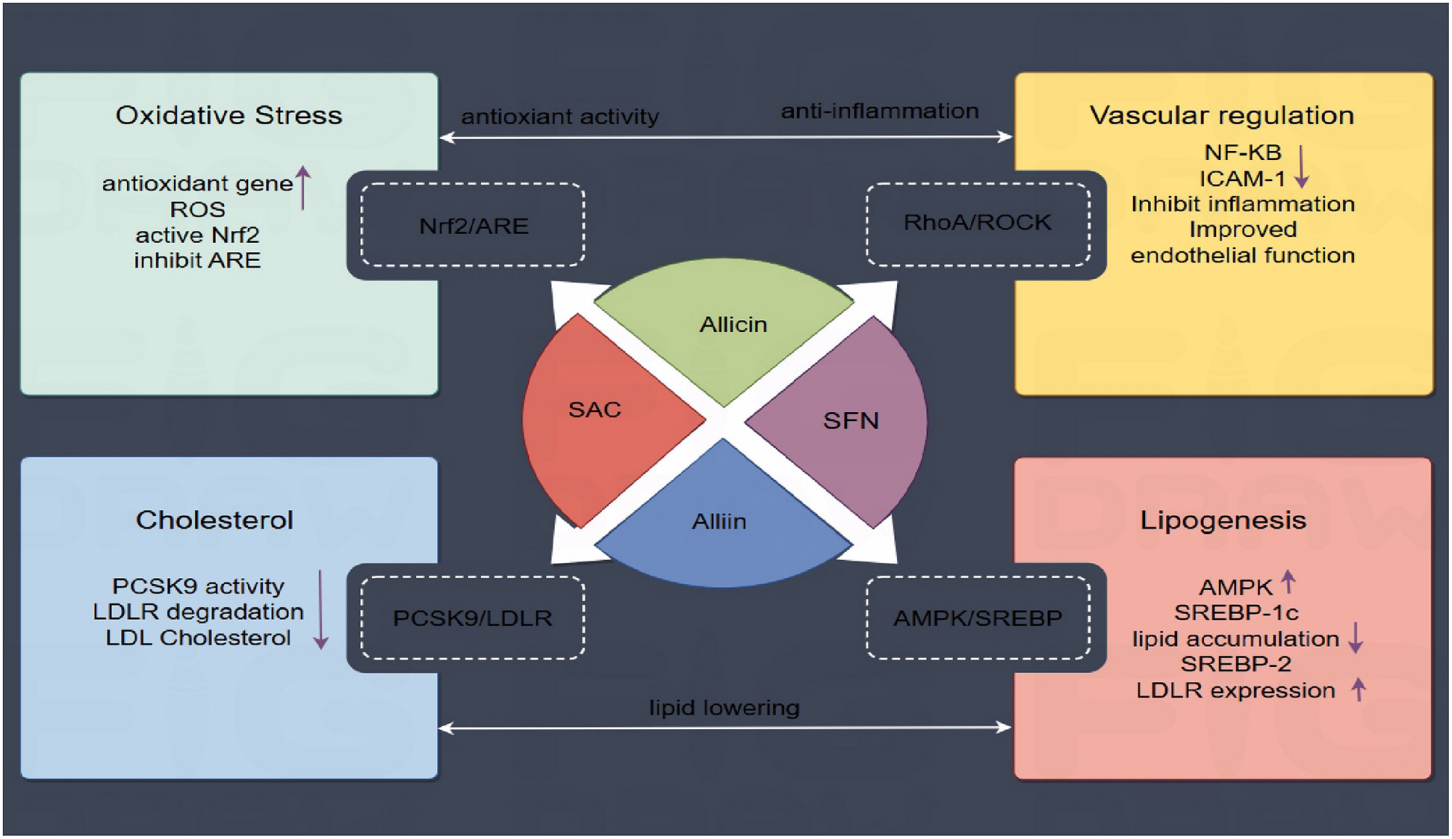
OSCs-Mediated signaling pathways in atherosclerosis prevention. OSCs regulate AS processes via various pathways. Four main mechanisms are shown: (1) Nrf2/ARE-mediated antioxidant response; (2) RhoA/ROCK vascular regulation; (3) AMPK/SREBP-controlled lipid metabolism; and (4) PCSK9 suppression of LDLR degradation.

To summarize, OSCs contribute to anti-atherosclerotic effects via modulating numerous crucial signaling pathways, primarily the four core mechanisms listed above, shown in Figure 4: (1) The Nrf2/ARE pathway represents an antioxidant pathway. OSCs, such as allicin and SAC, can directly activate Nrf2 resulting in antioxidant effects. (2) OSCs including SFN inhibit the RhoA/ROCK pathway, leading to improved endothelial function and reduced inflammation by downregulating NF-*κ*B. (3) The AMPK/SREBP-1c/SREBP-2 pathway is modulated by alliin, reducing lipid accumulation and cholesterol synthesis while promoting LDLR expression. (4) Alliin targets the PCSK9-LDLR pathway, inhibiting PCSK9 activity, limiting LDLR degradation and decreasing circulating LDL cholesterol. These multitarget mechanisms emphasize OSCs' potential for preventing AS by focusing on lipid metabolism, oxidative stress, inflammation, and endothelial dysfunction.

## 5 Conclusion

Natural organosulfur compounds, notably garlic derivatives, have been shown to have anti-atherogenic effects on several targets, including lipid metabolism, antioxidant and anti-inflammatory activity, endothelial function protection, and antibacterial capabilities. Their regulatory role on critical signaling pathways such as Nrf2, RhoA/ROCK, AMPK, and PCSK9 emphasizes their therapeutic potential. While preclinical evidence is robust, clinical translation remains hindered by challenges such as variable bioavailability, lack of standardized formulations, and insufficient large-scale human trials. Future research should concentrate on clinical trials to determine efficacy, increase bioavailability, and develop standardized OSCs formulations for cardiovascular disease prevention and management.
